# Dr. Harvey L. James

**Published:** 1903-05

**Authors:** 


Dr. Harvey L. James, of Shelby, N. Y., an alumnus of the University of Buffalo, class of 1881, died at his home March 14, 1903, aged 46 years. He had been ill for a long time, his disease being phthisis pulmonalis.

